# Treatment outcomes and HPV characteristics for an institutional cohort of patients with anal cancer receiving concurrent chemotherapy and intensity-modulated radiation therapy

**DOI:** 10.1371/journal.pone.0194234

**Published:** 2018-03-09

**Authors:** Corey C. Foster, Andrew Y. Lee, Larissa V. Furtado, John Hart, Lindsay Alpert, Shu-Yuan Xiao, Neil H. Hyman, Manish R. Sharma, Stanley Liauw

**Affiliations:** 1 Department of Radiation and Cellular Oncology, The University of Chicago Medicine, Chicago, Illinois, United States of America; 2 Department of Pathology, University of Utah/ARUP Laboratories, Salt Lake City, Utah, United States of America; 3 Department of Pathology, The University of Chicago Medicine, Chicago, Illinois, United States of America; 4 Department of Colon and Rectal Surgery, The University of Chicago Medicine, Chicago, Illinois, United States of America; 5 Section of Hematology/Oncology, Department of Medicine, The University of Chicago Medicine, Chicago, Illinois, United States of America; Massachusetts General Hospital, UNITED STATES

## Abstract

**Background:**

Intensity-modulated radiation therapy (IMRT) has been used to limit treatment-related toxicity for patients with anal squamous cell carcinoma (SCC). The treatment outcomes and HPV characteristics for a cohort of patients receiving definitive concurrent chemotherapy and IMRT are reported.

**Materials and methods:**

52 patients with anal SCC were treated with IMRT and concurrent chemotherapy. Radiation was delivered sequentially to the pelvis and inguinal lymph nodes (45 Gy) and anal tumor (median dose, 54 Gy). Multiplex real-time PCR for 7 high-risk HPV subtypes (n = 22) and p16 immunohistochemistry (n = 21, rated on a 0, 1, and 2+ scale) were performed on available specimens. Survival was estimated using Kaplan-Meier analysis, and toxicities were recorded.

**Results:**

Median follow-up was 33 months. Three-year freedom from locoregional failure (FFLRF), freedom from distant metastasis (FFDM), freedom from colostomy (FFC), and overall survival (OS) were 94%, 85%, 91%, and 90%, respectively. Acute grade 2+ skin, GI, and GU toxicities occurred in 83%, 71%, and 19% of evaluable patients, respectively. The rates of late grade 2+ GI and GU toxicities for evaluable patients (n = 32) were 28% and 9%, respectively. Of patients with available pathology, 91% and 71% were positive for HPV and p16 (2+), respectively. HPV genotypes included 16 (n = 17), 33 (n = 2), 18 (n = 1), and 45 (n = 1). HPV and p16 status were associated on Chi-square analysis (p = 0.07). Neither HPV nor p16 status was significantly associated with any clinical outcome. For HPV+ patients, 3-year FFLRF, FFDM, FFC, and OS were 100%, 69%, 100%, and 88%, respectively.

**Conclusions:**

In this patient cohort, disease control was excellent for anal SCC treated with definitive concurrent chemotherapy and IMRT, and treatment was well tolerated. HPV and p16 status were not prognostic for treatment outcomes which may be related to our small sample size.

## Introduction

Approximately 8,200 new diagnoses of anal cancer and 1,100 deaths from this disease are expected in the United States in 2017 [1]. Although it is considered an uncommon malignancy, the incidence of anal squamous cell carcinoma (SCC) increased at a rate of nearly 2.2%/year from 2005–2014 [2]. Historically, this disease was treated with abdominoperineal resection (APR) with associated 5-year overall survival (OS) ranging from 40–70% [3]. Given the morbidity accompanying APR, attempts at nonoperative management were a welcome paradigm shift since Nigro et al. reported complete tumor response in 23 of 28 patients receiving neoadjuvant concurrent fluorouracil (5FU), mitomycin (MMC), and radiation (RT) [4]. More recently, numerous randomized trials have established concurrent chemoradiation (CRT) as the standard of care for anal cancer [5–9]. While CRT is associated with 5-year OS of 50–75% and 5-year colostomy-free survival of 60–75%, it is not without toxicity [5–9]. For instance, rates of acute grade 3–4 toxicity for patients receiving RT+5FU/MMC in RTOG 9811 were 62% for hematologic toxicity, 49% for dermatologic toxicity, and 37% for GI toxicity [8].

One approach to reduce the toxicity associated with definitive CRT has been the use of intensity-modulated radiation therapy (IMRT). In a phase II, single-arm study, RTOG 0529 investigated the ability of dose-painted IMRT+5FU/MMC to reduce acute grade 2+ GI/GU toxicity compared to the standard arm of RTOG 9811 [10]. The primary endpoint was not met; however, IMRT was associated with a decreased risk of grade 2+ hematologic, grade 3+ gastrointestinal, and grade 3+ dermatologic events [10]. Secondary analyses have correlated this reduction in toxicity to reduced RT doses to the small bowel and anterior pelvic contents [11]. Moreover, multiple retrospective studies have suggested similar oncologic outcomes for patients treated with IMRT compared to patients receiving older RT techniques [12–15]. In this report, we update our institutional experience using a predominantly sequential-boost IMRT technique with concurrent chemotherapy for the definitive treatment of anal SCC.

Another evolving area of research in the field of anal carcinoma is investigation of the relationship between this disease and human papillomavirus (HPV) [16–18]. Previous reports have suggested that, similar to cervical cancer, the majority of anal SCCs are associated with HPV infection [16–18]. Specifically, the high-risk HPV subtype 16 has been implicated as the most commonly detected HPV genotype [17, 18]. However, several groups have studied the prognostic utility of HPV status in anal SCC with mixed results [19–23]. Interestingly, one such study investigating 143 tumors found p16-positivity to be independently associated with OS [21]. To be a clinically-useful indicator of HPV infection, the correlation between p16-positivity and actual HPV infection must be reliable, and at least one group has shown a strong correlation between p16 status and HPV detected by chromogenic in-situ hybridization (ISH) [22]. Herein, we analyzed the p16/HPV characteristics and prognostic ability of p16/HPV status for a subset of patients with anal SCC receiving definitive IMRT and chemotherapy.

## Materials and methods

### Patient selection

The medical records of 52 patients with anal SCC treated with definitive IMRT and concurrent chemotherapy at a single institution from 2000–2016 were retrospectively reviewed. The University of Chicago Institutional Review Board (IRB) approved of this study (IRB14-0099). The majority of patients provided written informed consent for inclusion in a long-term database for investigation of anal cancer outcomes and toxicity. Waiver of informed consent due to the retrospective nature of this work was obtained for all other patients for whom follow-up was not ongoing. The University of Chicago IRB approved both the written informed consent procedure and waiver of informed consent in patients who could not provide written informed consent.

Prior to treatment, all patients had a complete history and physical, workup as deemed appropriate by the treating physician, and staging according to the American Joint Committee on Cancer 7th Edition. Patients did not receive induction chemotherapy and all were treated with curative intent for non-metastatic disease. Of the 52 patients in the IMRT cohort, 22 had readily-available tumor specimens that were evaluated for HPV and p16 status. HPV genotype was performed using a multiplex, real-time polymerase chain reaction (PCR) assay. Specifically, this PCR detects seven clinically-relevant, high-risk HPV subtypes including HPV 16, 18, 31, 33, 45, 52, and 58. Tissue was also tested for the presence of p16 expression using immunohistochemistry (IHC) with intensity of staining rated on a 0, 1, and 2+ scale. A positive result was defined as 2+ staining.

### Treatment technique

IMRT most commonly consisted of a sequential-boost technique delivering 45 Gy to the pelvis (with a reduction after 30.6 Gy including only the low pelvis if node negative) and inguinal lymph nodes, and 54 Gy to the anal tumor in 1.8 Gy/fraction. Five patients received IMRT using a simultaneous-integrated boost technique in which involved lymph nodes >1.5 cm in size received 2.0–2.2 Gy/fraction over the first 25 days. Prospective bone marrow-sparing guidelines were observed in 20 patients. Concurrent chemotherapy consisted of 5FU/MMC in 87% of patients and 5FU/cisplatin in 10% of patients. Patients typically received 5FU 1000 mg/m2 by continuous intravenous infusion on days 1–4 and days 29–32 of IMRT, and MMC 10 mg/m2 intravenously on days 1 and 29 of IMRT. One patient received 5FU alone, and one patient received unknown chemotherapy. Toxicities were graded using the Common Terminology Criteria for Adverse Events, version 4, at weekly on-treatment visits while receiving IMRT and during regular follow-up after completion of therapy typically occurring at least every 6–12 months. Late toxicity was defined to occur 3 months after completion of IMRT.

### Endpoints and statistical analysis

Endpoints included freedom from locoregional failure (FFLRF), freedom from distant metastasis (FFDM), freedom from colostomy (FFC), and OS. All endpoints were calculated from the date of completion of IMRT. FFLRF was defined as the time to any local or regional failure detected on physical exam, colonoscopy, or imaging of the abdomen/pelvis. FFDM was defined as the time to development of any extrapelvic distant disease detected on imaging. FFC was defined as time to the date of the operation. OS was defined as the time to death from any cause as determined from review of medical records or the Social Security Death Index. Univariate survival analyses were estimated using Kaplan-Meier methods. Patients were censored as appropriate for death or loss to follow-up. Correlation between HPV detected via PCR and p16 detected via IHC was performed with Chi-square analysis. All statistics were performed using JMP Statistical Software (v 13.0, SAS Institute, Cary, NC).

## Results

### Patient characteristics

Patient demographics, tumor characteristics, and treatment details are displayed in Table 1. The median age was 57 years, and the majority of patients were female (71%) with T2/T3 (79%), N0 (62%) disease. Seven patients (14%) had a history of human immunodeficiency virus infection. All patients received concurrent chemotherapy most commonly consisting of 5FU/MMC (87%). The median prescription to the anal tumor was 54 Gy (range 45–59.4 Gy) delivered using a sequential-boost technique. Treatment breaks ranged from 1–10 days with 39% of patients requiring a break of any duration. Of patients experiencing treatment breaks, 85% had breaks at least 3 days long. The most common documented reasons for a treatment break were hematologic toxicity (40%) and skin toxicity (25%). The remaining patients had breaks for generalized complaints including weakness/dehydration (10%) or unknown reasons. The median treatment duration was 42 days (range 32–58) from the first to last fraction of IMRT.

**Table 1:** Patient and tumor characteristics.

Variable	No. of Patients (52 total)	%
**Age, years**		
Median	57	
Range	39–87	
**Sex**		
Male	15	28.8
Female	37	71.2
**HIV status**		
Positive	7	13.5
Negative	45	86.5
**Race/ethnicity**		
White	27	51.9
Black	21	40.3
Hispanic	2	3.8
Unknown	2	3.8
**T stage**		
TX	1	1.9
T1	6	11.5
T2	23	44.2
T3	18	34.6
T4	4	7.7
**N stage**		
NX	2	3.8
N0	32	61.5
N1	6	11.5
N2	4	7.7
N3	8	15.4
**HPV Status**		
Positive	20	38.5
Negative	2	3.8
Unknown	30	67.7
**Chemotherapy**		
**5FU**^**a**^**/MMC**^**b**^	45	86.5
5FU/cisplatin	5	9.6
Other	1	1.9
Unknown	1	1.9
**RT**^**c**^ **dose, Gy**		
Median	54	
Range	45–59.4	
**RT break**		
Yes	20	38.5
Break range, days	1–10	
No	32	61.5

^a^fluorouracil

^b^mitomycin

^c^radiation

### HPV characteristics

Twenty-two tumor samples were tested for HPV genotype, and 21 were tested for p16 positivity. HPV was detected via PCR in 91% of samples. HPV genotypes included 16 (80%), 33 (10%), 18 (5%), and 45 (5%). Additionally, IHC staining was positive for p16 in 71% of tested samples. On Chi-square analysis, p16 and HPV status were associated with one another (p = 0.07).

### Treatment-related toxicity

Acute toxicity data are listed in Table 2. Of patients with available toxicity data, acute grade 2+ skin, gastrointestinal (GI), and genitourinary (GU) toxicities occurred in 94%, 81%, and 19% of patients, respectively. Additionally, grade 3+ acute skin, GI, and GU toxicities occurred in 48%, 7%, and 0% of evaluable patients, respectively. Acute hematologic toxicity data were available on less than half of the cohort. Of note, grade 3+ leukopenia was experienced by 62.5% of evaluable patients (n = 15/24). Late GI and GU toxicity data were available for 62% of patients (n = 32). Late grade 2 and grade 3 GI toxicity occurred in 25% (n = 8/32) and 3% (n = 1/32) of evaluable patients, respectively, whereas late grade 2 GU toxicity occurred in 9% of evaluable patients (n = 3/32). No evaluable patients experienced late grade 3+ GU toxicity.

**Table 2:** Maximum acute toxicity.

Toxicity	Grade 2 No. (%)	Grade 3 No. (%)	Grade 4 No. (%)	Unknown No. (%)
**Skin**	21 (40.4%)	22 (42.3%)	0 (0%)	6 (11.5%)
**GI**	34 (65.4%)	3 (5.8%)	0 (0%)	6 (11.5%)
**GU**	10 (19.2%)	0 (0%)	0 (0%)	11 (21.1%)
**Leukopenia**	5 (9.6%)	12 (23.1%)	3 (5.8%)	28 (53.8%)
**Neutropenia**	7 (13.5%)	7 (13.5%)	5 (9.6%)	30 (57.7%)
**Anemia**	7 (13.5%)	3 (5.8%)	0 (0%)	28 (53.8%)
**Thrombocytopenia**	5 (9.6%)	5 (9.6%)	1 (1.9%)	30 (57.7%)

### Treatment outcomes and failures

**Fig 1:**
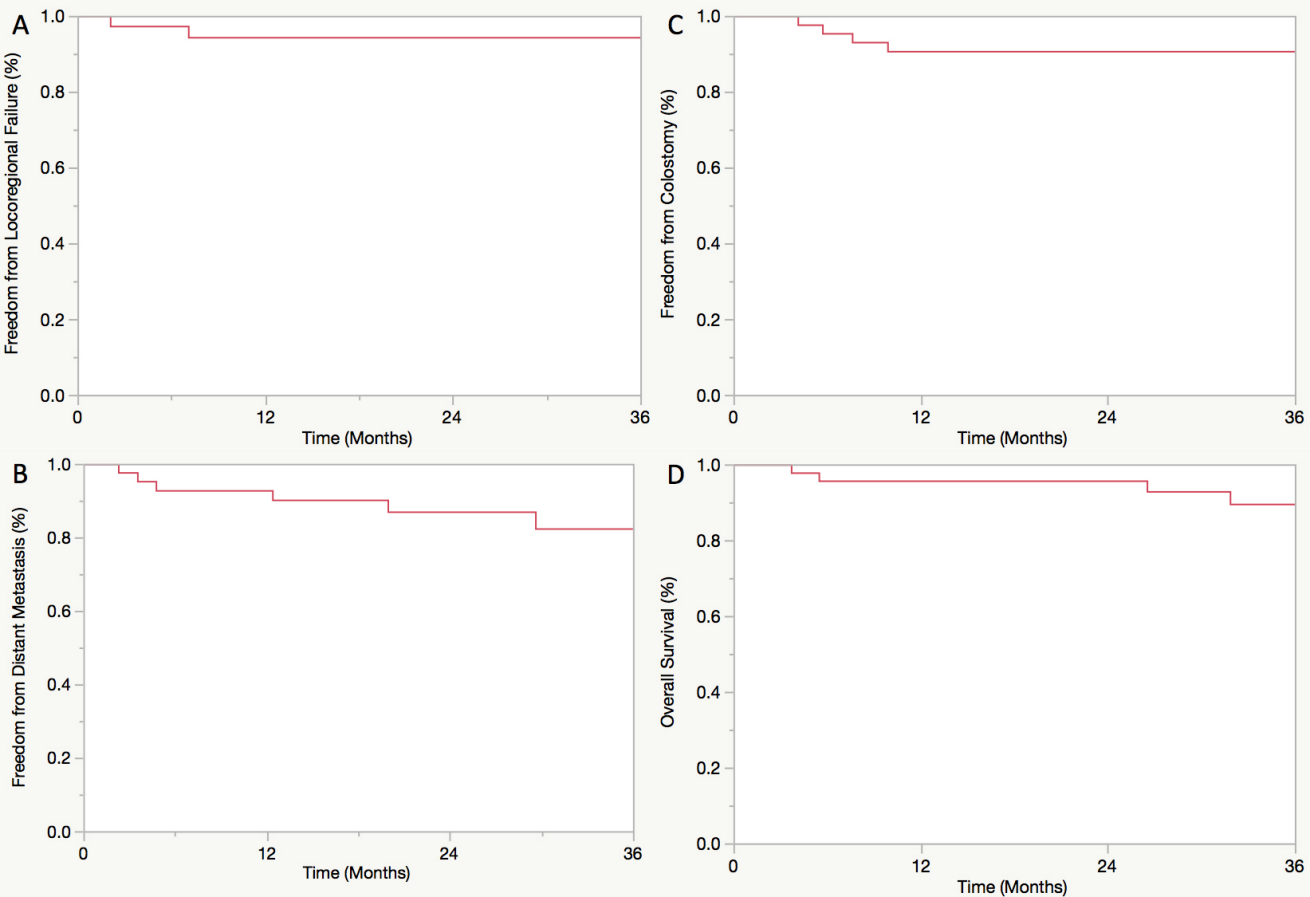
Freedom from locoregional failure (A), freedom from distant metastasis (B), freedom from colostomy (C), and overall survival (D) for all patients receiving IMRT with concurrent chemotherapy.

**Fig 2:**
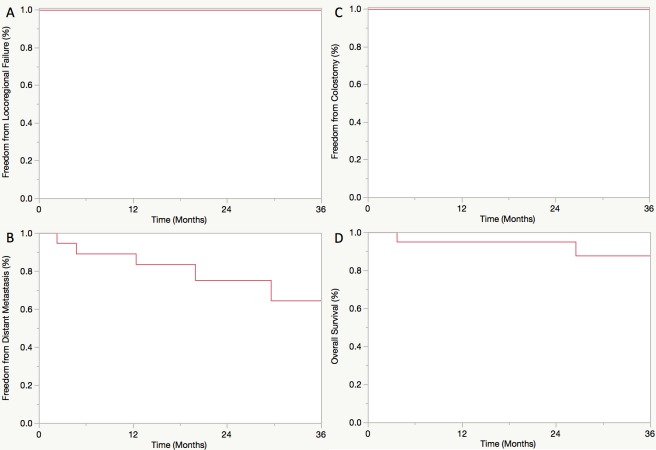
Freedom from locoregional failure (A), freedom from distant metastasis (B), freedom from colostomy (C), and overall survival (D) for patients with HPV+ disease.

Median follow-up was 33 months from completion of IMRT. Kaplan-Meier survival curves displaying treatment outcomes for all patients receiving IMRT with concurrent chemotherapy (n = 52) and the subset of HPV+ patients (n = 20) are displayed in Figs 1 and 2, respectively. Three-year estimated FFLRF, FFDM, FFC, and OS in the entire cohort were 94%, 85%, 91%, and 90%, respectively. Of patients experiencing locoregional failure (6%), one experienced local failure, one experienced regional failure, and one experienced combined local and regional failure. The interval to locoregional failure ranged from 2–20 months after completion of IMRT. For patients experiencing distant failure (n = 5), the interval to detection of metastatic disease from completion of IMRT ranged from 2–12 months. Four of 5 patients experiencing DM had HPV+ disease with the remaining patient having unknown HPV status. One HPV+ patient experienced oligometastatic DM manifesting as a single right lower lobe pulmonary nodule. She underwent wedge resection and continued to have no evidence of disease 2 years postoperatively throughout her last follow-up. Colostomy was performed on a total of 4 patients. The indication for colostomy was salvage of local recurrence of anal SCC in 2 patients, prophylactic colostomy for history of familial adenomatous polyposis in 1 patient, and resection of a metachronous colorectal cancer in 1 patient. For HPV+ patients, 3-year FFLRF, FFDM, FFC, and OS were estimated to be 100%, 69%, 100%, and 88%, respectively. On univariate analysis using Kaplan-Meier methods, neither HPV status nor p16 status was significantly associated with any endpoint (all p>0.05), although there was a trend towards improved OS for p16+ patients (p = 0.09).

## Discussion

In this report with a median follow-up of 33 months, we update our institutional outcomes and toxicity data for patients with anal SCC receiving concurrent chemotherapy and IMRT at time points approximately twice as long after completion of IMRT compared to our previously published experience [13]. As long-term outcomes and toxicity data have not been prospectively reported for IMRT for anal cancer, our study adds to a growing body of retrospective literature on this topic [12–15]. Additionally, we performed HPV genotyping and p16 IHC for a subset of tumor specimens to explore the HPV characteristics of our patient population. Three-year locoregional disease control was excellent for the overall cohort and 100% for a subset of patients with HPV+ disease. In general, concurrent chemotherapy and IMRT was well-tolerated with patients most frequently experiencing acute grade 2+ dermatologic and GI toxicity. Moreover, the majority of patients with known HPV status were found to have HPV+ disease (91%) with the most common HPV genotype being HPV 16 (80%).

Overall, outcomes for our cohort were superior or comparable to the long-term outcomes for patients receiving RT+5FU/MMC on RTOG 9811, although differences in patient populations as well as reporting of outcomes from time of randomization in RTOG 9811 rather than from time of completion of RT as in our study limit direct comparison [8]. For our entire cohort, 3-year FFLRF, FFDM, and OS were 94%, 85%, and 90%, respectively. The same 5-year outcomes from RTOG 9811 were 80%, 87%, and 78%. These favorable long-term IMRT outcomes were associated with a reasonable toxicity profile, although toxicity data may be under-reported given the retrospective nature of this report. Our 7% rate of acute grade 3+ GI toxicity compares quite favorably to the rates for the same toxicity in the standard arm of RTOG 9811 (36%) and in RTOG 0529 (21%). On the other hand, the risk of acute grade 3+ skin toxicity (48%) was comparable to RTOG 9811 (49%) and significantly higher than the 21% rate reported in RTOG 0529. It is possible that differences in IMRT technique may have contributed to this discrepancy, especially considering the variation in target delineation across clinicians found on central review in RTOG 0529. Additionally, our IMRT approach relied predominantly on a sequential-boost technique rather than dose-painting. Hematologic toxicity was evaluable for less than half of our cohort with 19 of 22 evaluable patients (86.3%) experiencing grade 2+ neutropenia. This is relatively similar to the rate of any grade 2+ hematologic toxicity in RTOG 9811 (85%) and higher than the same rate reported for RTOG 0529 (73%). In the future, routine use of bone marrow-sparing dosimetric constraints may more effectively limit hematologic toxicity with IMRT [24].

We also report the HPV characteristics for a subset of patients with anal SCC receiving IMRT with concurrent CRT. Consistent with other patient cohorts, our population had predominantly HPV+ tumors with the most common HPV genotype being HPV 16 [16–18]. Of note, HPV status detected by PCR and p16 IHC were associated on Chi-square analysis. This suggests that IHC may be a sufficient screening tool that can be performed more quickly and more cost-effectively than PCR. However, caution is needed when considering the routine adoption of this approach clinically as stratification using combined HPV and p16 status has been associated with improved prognostication compared to using either characteristic alone [19]. Specifically, patients with HPV-/p16+ disease were noted to have 5-year local control of just 63.6% compared to 88.1% for patients with HPV+/p16+ disease in one German cohort [19]. This may be related to the unique biology of p16 overexpression that may develop independently of HPV infection, as has been observed in the setting of oropharyngeal SCC [25]. Our excellent locoregional control for patients with HPV+ anal SCC is also consistent with multiple reports from other institutions [19, 21–22]; however, our small sample size with only 2 patients having confirmed HPV- disease limits our ability to draw conclusions regarding the prognostic significance of HPV status. Our original study hypothesis that HPV+ patients may be preferential candidates for treatment de-escalation is therefore not supported by the data, although it is fair to consider de-intensification as a justifiable topic of study given the very high rate of local control for all patients treated with 54 Gy and 5FU/MMC. The seminal study by Nigro et al. reported an 82% pathologic complete response rate for patients receiving only 30 Gy in 2 Gy/fraction with concurrent 5FU/MMC; however, only 7% of patients in this series had node-positive disease [4].

Despite our high rate of FFLRF for all patients and the subset with known HPV+ disease, the risk of distant failure continued to be substantial with 3-year rates of distant metastasis of 15% for the entire cohort and 31% for those with known HPV+ disease. This predilection for systemic failure was also suggested by Yhim et al. who reported that patients with HPV+ anal SCC had superior locoregional oncologic outcomes but had similar risk of systemic failure compared to HPV- patients [22]. Therefore, future improvements in disease control for anal SCC may rely on new chemotherapeutic approaches such as the selective use of adjuvant systemic therapy for patients at high-risk for development of metastatic disease. Aggressive treatment of oligometastatic disease may also be beneficial with one patient in our cohort experiencing no progression of disease nearly 2 years after wedge resection of an isolated pulmonary metastasis. The benefits of adopting a treatment paradigm of local ablation for oligometastatic disease have recently been reported for other primary malignancies most notably including non-small-cell lung cancer [26].

## Conclusions

Although the present study is limited by its retrospective nature with limited evaluable pathology and follow-up data, it adds to a growing body of literature investigating HPV characteristics for patients with anal cancer and the role of IMRT in treatment of this disease. Definitive CRT will likely continue to rely on IMRT to decrease toxicity of locoregional therapy without compromising tumor control. Further studies analyzing the relationship between HPV status and oncologic outcomes for anal cancer are warranted.

## References

[1] Siegel RL, Miller KD, Jemal A. Cancer Statistics, 2017. CA Cancer J Clin. 2017;67: 7–30. doi: 10.3322/caac.21387 28055103 10.3322/caac.21387

[2] Howlander N, Noone AM, Krapcho M, Miller D, Bishop K, Kosary CL, et al. SEER Cancer Statistics Review, 1975–2014. c2017 [cited 2018 Feb 6]. Available from: https://seer.cancer.gov/statfacts/html/anus.html

[3] Ryan DP, Compton CC, Mayer RJ. Carcinoma of the anal canal. N Engl J Med. 2000;342: 792–800. doi: 10.1056/NEJM200003163421107 10717015 10.1056/NEJM200003163421107

[4] Nigro ND, Seydel HG, Considine B, Vaitkevicius VK, Leichman L, Kinzie JJ. Combined preoperative radiation and chemotherapy for squamous cell carcinoma of the anal canal. Cancer. 1983;51: 1826–9. 6831348 10.1002/1097-0142(19830515)51:10<1826::aid-cncr2820511012>3.0.co;2-l

[5] Northover J, Glynne-jones R, Sebag-Montefiore D, James R, Meadows H, Wan S, et al Chemoradiation for the treatment of epidermoid anal cancer: 13-year follow-up of the first randomised UKCCCR Anal Cancer Trial (ACT I). Br J Cancer. 2010;102: 1123–8. doi: 10.1038/sj.bjc.6605605 20354531 10.1038/sj.bjc.6605605 PMC2853094

[6] Bartelink H, Roelofsen F, Eschwege F, Rougier P, Bosset JF, Gonzalez DG, et al Concomitant radiotherapy and chemotherapy is superior to radiotherapy alone in the treatment of locally advanced anal cancer: results of a phase III randomized trial of the European Organization for Research and Treatment of Cancer Radiotherapy and Gastrointestinal Cooperative Groups. J Clin Oncol. 1997;15: 2040–9. doi: 10.1200/JCO.1997.15.5.2040 9164216 10.1200/JCO.1997.15.5.2040

[7] Flam M, John M, Pajak TF, Petrelli N, Myerson R, Doggett S, et al Role of mitomycin in combination with fluorouracil and radiotherapy, and of salvage chemoradiation in the definitive nonsurgical treatment of epidermoid carcinoma of the anal canal: results of a phase III randomized intergroup study. J Clin Oncol. 1996;14: 2527–39. doi: 10.1200/JCO.1996.14.9.2527 8823332 10.1200/JCO.1996.14.9.2527

[8] Gunderson LL, Winter KA, Ajani JA, Pedersen JE, Moughan J, Benson AB 3rd, et al Long-term update of US GI intergroup RTOG 98–11 phase III trial for anal carcinoma: survival, relapse, and colostomy failure with concurrent chemoradiation involving fluorouracil/mitomycin versus fluorouracil/cisplatin. J Clin Oncol. 2012;30: 4344–51. doi: 10.1200/JCO.2012.43.8085 23150707 10.1200/JCO.2012.43.8085 PMC3515768

[9] James RD, Glynne-Jones R, Meadows HM, Cunningham D, Myint AS, Saunders MP, et al Mitomycin or cisplatin chemoradiation with or without maintenance chemotherapy for treatment of squamous-cell carcinoma of the anus (ACT II): a randomised, phase 3, open-label, 2 × 2 factorial trial. Lancet Oncol. 2013;14: 516–24. doi: 10.1016/S1470-2045(13)70086-X 23578724 10.1016/S1470-2045(13)70086-X

[10] Kachnic LA, Winter K, Myerson RJ, Goodyear MD, Willins J, Esthappan J, et al RTOG 0529: a phase 2 evaluation of dose-painted intensity modulated radiation therapy in combination with 5-fluorouracil and mitomycin-C for the reduction of acute morbidity in carcinoma of the anal canal. Int J Radiat Oncol Biol Phys. 2013;86: 27–33. doi: 10.1016/j.ijrobp.2012.09.023 23154075 10.1016/j.ijrobp.2012.09.023 PMC3619011

[11] Olsen JR, Moughan J, Myerson R, Abitbol A, Doncals DE, Johnson D, et al Predictors of radiation therapy-related gastrointestinal toxicity from anal cancer dose-painted intensity modulated radiation therapy: secondary analysis of NRG Oncology RTOG 0529. Int J Radiat Oncol Biol Phys. 2017;98: 400–408. doi: 10.1016/j.ijrobp.2017.02.005 28463160 10.1016/j.ijrobp.2017.02.005 PMC5639877

[12] Milano MT, Jani AB, Farrey KJ, Rash C, Heimann R, Chmura SJ. Intensity-modulated radiation therapy (IMRT) in the treatment of anal cancer: toxicity and clinical outcome. Int J Radiat Oncol Biol Phys. 2005;63: 354–61. doi: 10.1016/j.ijrobp.2005.02.030 16168830 10.1016/j.ijrobp.2005.02.030

[13] Salama JK, Mell LK, Schomas DA, Miller RC, Devisetty K, Jani AB, et al Concurrent chemotherapy and intensity-modulated radiation therapy for anal canal cancer patients: a multicenter experience. J Clin Oncol. 2007;25: 4581–6. doi: 10.1200/JCO.2007.12.0170 17925552 10.1200/JCO.2007.12.0170

[14] Call JA, Prendergast BM, Jensen LG, Ord CB, Goodman KA, Jacob R, et al Intensity-modulated radiation therapy for anal cancer: results from a multi-institutional retrospective cohort study. Am J Clin Oncol. 2016;39: 8–12. doi: 10.1097/COC.0000000000000009 24401669 10.1097/COC.0000000000000009 PMC10865428

[15] Mitra D, Hong TS, Horick N, Rose B, Drapek LN, Blaszkowsky LS, et al Long-term outcomes and toxicities of a large cohort of anal cancer patients treated with dose-painted IMRT per RTOG 0529. Adv Radiat Oncol. 2017;2: 110–117. doi: 10.1016/j.adro.2017.01.009 28740921 10.1016/j.adro.2017.01.009 PMC5514246

[16] Frisch M, Glimelius B, van den Brule AJ, Wohlfahrt J, Meijer CJ, Walboomers JM, et al Sexually transmitted infection as a cause of anal cancer. N Engl J Med. 1997;337: 1350–8. doi: 10.1056/NEJM199711063371904 9358129 10.1056/NEJM199711063371904

[17] Hoots BE, Palefsky JM, Pimenta JM, Smith JS. Human papillomavirus type distribution in anal cancer and anal intraepithelial lesions. Int J Cancer. 2009;124: 2375–83. doi: 10.1002/ijc.24215 19189402 10.1002/ijc.24215

[18] Ouhoummane N, Steben M, Coutlée F, Vuong T, Forest P, Rodier C, et al Squamous anal cancer: patient characteristics and HPV type distribution. Cancer Epidemiol. 2013;37: 807–12. doi: 10.1016/j.canep.2013.09.015 24139594 10.1016/j.canep.2013.09.015

[19] Mai S, Welzel G, Ottstadt M, Lohr F, Severa S, Prigge ES, et al Prognostic relevance of HPV infection and p16 overexpression in squamous cell anal cancer. Int J Radiat Oncol Biol Phys. 2015;93: 819–27. doi: 10.1016/j.ijrobp.2015.08.004 26530750 10.1016/j.ijrobp.2015.08.004

[20] Meyer JE, Panico VJ, Marconato HM, Sherr DL, Christos P, Pirog EC. HIV positivity but not HPV/p16 status is associated with higher recurrence rate in anal cancer. J Gastrointest Cancer. 2013;44: 450–5. doi: 10.1007/s12029-013-9543-1 24014082 10.1007/s12029-013-9543-1 PMC3963822

[21] Serup-Hansen E, Linnemann D, Skovrider-Ruminski W, Høgdall E, Geertsen PF, Havsteen H. Human papillomavirus genotyping and p16 expression as prognostic factors for patients with American Joint Committee on Cancer stages I to III carcinoma of the anal canal. J Clin Oncol. 2014;32: 1812–7. doi: 10.1200/JCO.2013.52.3464 24821878 10.1200/JCO.2013.52.3464

[22] Yhim HY, Lee NR, Song EK, Kwak JY, Lee ST, Kim JS, et al The prognostic significance of tumor human papillomavirus status for patients with anal squamous cell carcinoma treated with combined chemoradiotherapy. Int J Cancer. 2011;129: 1752–60. doi: 10.1002/ijc.25825 21128253 10.1002/ijc.25825

[23] Roldán Urgoiti GB, Gustafson K, Klimowicz AC, Petrillo SK, Magliocco AM, Doll CM. The prognostic value of HPV status and p16 expression in patients with carcinoma of the anal canal. PLoS ONE. 2014;9:e108790 doi: 10.1371/journal.pone.0108790 25271758 10.1371/journal.pone.0108790 PMC4182745

[24] Lee AY, Golden DW, Bazan JG, Kopec M, Pelizzari CA, Aggarwal S. Hematologic nadirs during chemoradiation for anal cancer: temporal characterization and dosimetric predictors. Int J Radiat Oncol Biol Phys. 2017;97: 306–312. doi: 10.1016/j.ijrobp.2016.10.010 28068238 10.1016/j.ijrobp.2016.10.010

[25] Allen CT, Lewis JS Jr, El-Mofty SK, Haughey BH, Nussenbaum B. Human papillomavirus and oropharynx cancer: biology, detection and clinical implications. Laryngoscope. 2010;120: 1756–72. doi: 10.1002/lary.20936 20669304 10.1002/lary.20936

[26] Gomez DR, Blumenschein GR Jr, Lee JJ, Hernandez M, Ye R, Camidge DR, et al Local consolidative therapy versus maintenance therapy or observation for patients with oligometastatic non-small-cell lung cancer without progression after first-line systemic therapy: a multicentre, randomised, controlled, phase 2 study. Lancet Oncol. 2016;17: 1672–1682. doi: 10.1016/S1470-2045(16)30532-0 27789196 10.1016/S1470-2045(16)30532-0 PMC5143183

