# Correction: Treatment outcomes and HPV characteristics for an institutional cohort of patients with anal cancer receiving concurrent chemotherapy and intensity-modulated radiation therapy

**DOI:** 10.1371/journal.pone.0200400

**Published:** 2018-07-05

**Authors:** Corey C. Foster, Andrew Y. Lee, Larissa V. Furtado, John Hart, Lindsay Alpert, Shu-Yuan Xiao, Neil H. Hyman, Manish R. Sharma, Stanley L. Liauw

The ninth author’s name is missing the middle initial. The correct name is: Stanley L. Liauw.
